# Mycobiota, neuro-cognitif disorders and behavioural impairments: is there a relationship?

**DOI:** 10.1192/j.eurpsy.2023.760

**Published:** 2023-07-19

**Authors:** B. Abdelmoula, H. Sellami, S. Neji, M. Torjmen, N. Bouayed Abdelmoula

**Affiliations:** 1 Genomics of Signalopathies at the service of Medicine; 2 Mycology Drosophila research unit, Medical University of Sfax; 3 Department of informatics, The National Engineering School of Sfax, Sfax, Tunisia

## Abstract

**Introduction:**

The human body carries large and diverse communities of symbiotic microbes that are important for human health and development. While the impact of the bacterial microbiota, which are mostly found in the human gut, on host physiology is relatively well described, much less is known about the interactions between the mycobiota and the host and the resulting effects on human health. At the level of the nervous system, there is increasing evidence implicating the gut microbiota in a variety of neurological disorders. Similar demonstrations of a causal or supportive role of the mycobioma in neurological disorders are still rare, but several studies linking fungal dysbiosis to disease in humans suggest a contribution of symbiotic fungi to neurocognitive and behavioral disorders.

**Objectives:**

We aim through this review to show the role of mycobiota in neurocognitive and behavioral disorders.

**Methods:**

We comprehensively review the scientific literature using Pubmed database and other search platforms such as Google scholar to state the role of mycobiota in neurocognitive and behavioral disorders.

**Results:**

Our bibliographic review revealed that, according to recent studies, Candida species are overrepresented in the stool of individuals with autism spectrum disorders and Rett syndrome compared to healthy controls. Other studies revealed mycobiome signatures specific to cognitive impairment and demonstrated that different diets modulate the mycobiome in association with Alzheimer’s disease markers and fungal-bacterial co-regulatory networks in patients with cognitive impairment.

**Conclusions:**

Our understanding of the role of the mycobiota in the biology of neurocognitive disorders-whether causal, consequential, or predisposing-could open up new hypotheses in this area and inspire further research on potential mycobiotic signatures, associated dysbiosis and dysfunction in the neurocognitive developmental-homeostasis spectrum that may contribute to neurocognitive and behavioral developmental disorders and predisposition to cognitive decline, dementia, and progression of neurodegenerative diseases including Parkinson’s and Alzheimer’s disease in high-risk subjects.

**Disclosure of Interest:**

None Declared

